# Capturing, clarifying, and consolidating the curiosity-creativity connection

**DOI:** 10.1038/s41598-022-19694-4

**Published:** 2022-09-12

**Authors:** Wilma Koutstaal, Kara Kedrick, Joshua Gonzalez-Brito

**Affiliations:** Department of Psychology, University of Minnesota, Minneapolis, MN 55455 USA

**Keywords:** Psychology, Human behaviour

## Abstract

The cognitive-motivational concepts of curiosity and creativity are often viewed as intertwined. Yet, despite the intuitively strong linkage between these two concepts, the existing cognitive-behavioral evidence for a curiosity-creativity connection is not strong, and is nearly entirely based on self-report measures. Using a new lab-based Curiosity Q&A task we evaluate to what extent behaviorally manifested curiosity—as revealed in autonomous inquiry and exploration—is associated with creative performance. In a preregistered study (*N* = 179) we show that, as hypothesized, the novelty of the questions that participants generated during the Curiosity Q&A Task significantly positively correlated with the originality of their responses on a divergent-thinking task (the conceptually-based Alternative Uses Task). Additionally, the extent to which participants sought out information that was implicitly missing in the presented factual stimuli ("gap-related information foraging") positively correlated with performance on two predominantly convergent-thinking tasks (the Remote Associates Task and Analogy Completion). Question asking, topic-related information foraging, and creative performance correlated with trait-based "interest-type" curiosity oriented toward exploration and novelty, but not with "deprivation-type" curiosity focused on dispelling uncertainty or ignorance. Theoretically and practically, these results underscore the importance of continuing to develop interventions that foster *both* creative thinking and active autonomous inquiry.

## Introduction

It is almost considered a commonplace that curiosity goes hand-in-hand with creativity. It seems but a short step from our concept of *curiosity*—a desire to know, typified by such traits and activities as inquisitiveness, the exploration of novelty, and questioning^1–3^, to our concept of *creativity*, an exploratory endeavor involving the generation of original, useful, and valuable ideas, processes, or products^4–6^. The meanings of the two concepts seem to shade and blend, one with the other. Yet, despite our intuitive conjoining of curiosity with creativity, the empirical evidence supporting such an association is neither as direct, nor as probative, as we might initially suppose^7,8^.

At first glance, a 2019 meta-analysis of 10 studies, including data from more than 2600 individuals^9^, could be taken as securely documenting a strong positive association between the two constructs. The study reported a weighted effect size* r* of 0.41 between curiosity and creativity, with a 95% confidence interval {0.27, 0.54} for which the lower bound was a comfortable distance from zero. But a closer look uncovers a rather different picture. Much of the apparent robust strength of this association was attributable to comparing self-reports of creativity with self-reports of curiosity. Exclusive reliance on measures of self-report is well-known to be inferentially problematic on multiple counts, such as liability to socially-desirable responding, self-verification biases, influences arising from respondent's implicit theories about the covariation of traits, behaviors, or outcomes, and the potential inaccessibility of nonconscious cognitive-motivational processes^10–13^. When, instead, consideration was confined to the subset of studies that assessed the relation between curiosity and judge-rated or performance-based creativity—rather than self-assessed creativity—the effect size was markedly smaller: weighted effect size *r* = 0.16, 95% CI {0.10, 0.22}.

Equally important, every study in the meta-analysis relied on self-reports of *curiosity.* The meta-analysis is, then, entirely silent on the question of whether behaviorally-assessed curiosity and behaviorally-assessed creativity are positively associated, or on the strength of that association. Studies that reported positive associations between self-reports of trait curiosity and creative self-efficacy^14,15^ or between subjective trait-ratings of curiosity and performance-based creativity^16,17^ do not address the question: Is there a positive relation between behaviorally-based evaluations of creative performance and behaviorally-based measures of curiosity? If so, how strong is that relation, and how consistent is it across different ways of operationalizing or evaluating either construct?

The current research addresses this gap. We ask: How strong is the curiosity-creativity connection when *both constructs* are defined not by self-report but by independently assessed and quantified cognitive-behavioral measures? To what extent is curiosity—as evaluated by actually observed lab-based behaviors involving autonomous inquiry and exploration—associated with creative performance on lab-based tasks requiring the generation of original, varied, and creative ideas?

A nomological network approach to individual differences and cognitive process factors highlights several reasons we would expect to find that curiosity-related behaviors, such as the asking of questions^18,19^ and autonomously seeking information^20^, would be positively linked to creativity^21,22^. One prominent construct relevant here is that of openness to experience. Openness to experience—involving a cognitive-motivational disposition to flexibly and receptively approach novel ideas—is amongst the most consistently observed personality characteristics associated with creativity^23,24^. Notably, questionnaire-based assessments of openness to experience often tap into cognitive-behavioral predispositions related to curiosity, such as seeking to learn new information or exploring novel stimuli, ideas, or cultures. A recent factor analysis of the lower-level structure of the openness construct including items from 36 different openness-related scales, identified Curiosity as one of six facets of the overall trait, with individuals scoring high on this facet characterized as "inquisitive, perceptive," as having "a thirst or desire for knowledge" and as "interested in why and how things happen" (p. 35) [^25^, see also^26^].

Additional reasons to expect a positive relation between curiosity and creativity are provided by process-based theoretical approaches to creativity. In process-based views, the initial impetus for a creative endeavor may be highly similar to curiosity. Creative endeavors are often launched with such "information prospecting" actions as the motivated gathering of information, newly or uniquely identifying a problem, and combining or reorganizing extant knowledge structures^27–29^. Similarly, there are conceptual links from cognitive, computational, and educational perspectives on active self-directed learning, such as open inquiry and discovery-focused inquiry^30^, to cognitive search processes essential to innovation, including exploring information from varied sources and generating multiple potential ideas to solve a problem^31–33^. Indeed, one of the very few studies to experimentally induce curiosity and then to examine the downstream effects of such state curiosity on creative behavior found that inducing specific curiosity about a particular puzzle was later associated with heightened creative solutions to that puzzle^34^.

In the current report—including an initial smaller pilot study and a larger preregistered conceptual replication (https://osf.io/9cbxs)—we systematically examine the relation between two new behaviorally-based measures of curiosity, obtained using our recently developed Curiosity Q&A Task^35^ and several performance-based measures of creativity. The behavioral assessments of curiosity were independent of the creativity measures—allowing an evaluation of the curiosity-creativity connection in a context uninfluenced by an individual's prior task-specific interest or prior attentional-motivational investment in the same task.

In both the pilot study and the preregistered study, we assessed curiosity in three primary ways: (1) the number and novelty of topic-related questions that participants generated in response to encountering various factual statements presented in the Curiosity Q&A Task; (2) the extent to which participants autonomously sought-out information that was implicitly missing or that implied a knowledge "gap"^22^ in those statements ("gap-related information foraging") or engaged in more general exploration of the topic ("topic-related information foraging"); and (3) standardized questionnaires of trait curiosity and the curiosity-based facet of openness to experience, included to allow examination of proposed subtypes of curiosity (explained below) that might differentially correlate with creative ideation.

Creativity was similarly assessed in multiple ways. Given the centrality of divergent thinking—that is, the ability to flexibly generate a plurality of varied novel ideas in response to a comparatively open-ended prompt^36,37^—to the creative process, we included two different assessments of predominantly divergent thinking. These included participants' responses to (1) the often used conceptually-prompted Alternative Uses Task^38,39^; and (2) a recently developed perceptually-based ambiguous object construal task, the "Figural Interpretation Quest"^40–42^. For each of these divergent tasks we assessed the originality of the ideas participants generated, the number of ideas they gave (i.e., fluency), and the variety of the ideas they generated across different semantic or conceptual categories (i.e., flexibility). Furthermore, recognizing that the creative process is widely viewed as also calling on aspects of convergent thinking, where one or a few optimal problem-solving solutions must be identified that aptly meet specified constraints^37,43^, we also included two different assessments that predominantly (although not exclusively) call upon convergent ideation, including (3) the Remote Associates Task^44^, and (4) (in the main study only) an Analogy Completion task^45^.

Some researchers^46–48^ have additionally differentiated between curiosity that is broadly oriented toward exploration and novelty (termed "Interest-type curiosity" or "Joyous Exploration"), as opposed to curiosity that is somewhat more narrowly oriented toward acquiring information that is missing from one's current knowledge or that is needed to solve a particular problem (termed "Deprivation-type" curiosity or "Deprivation Sensitivity"). As its name suggests, "Joyous Exploration" is characterized as a diversive, exploratory form of curiosity that involves the anticipated pleasure and enjoyment of learning new ideas or concepts (e.g., "I find it fascinating to learn new information"; "I enjoy learning about subjects that are unfamiliar to me"). This type of curiosity may map most closely to curiosity based on novelty, and the long-standing proposal that gaining information about novel stimuli is intrinsically rewarding^32,49^. In contrast, deprivation-type curiosity is more focused on particular instances of problem solving, and a desire to reduce uncertainty or remove ignorance (e.g., "I can spend hours on a single problem because I just can't rest without knowing the answer"; "I feel frustrated if I can't figure out the solution to a problem, so I work even harder to solve it"). This type of curiosity may map to an alternative construal of curiosity that emphasizes not novel stimuli but, instead, complex partially familiar stimuli about which the individual already has moderate knowledge—but that knowledge can be continually extended to progressively remove uncertainty. For example, in the "learning progress account" of curiosity^50,51^ acquiring further knowledge is intrinsically rewarding because "the brain is intrinsically motivated to pursue tasks in which one's predictions are always improving"^32^ (p. 457). To provide a more fine-grained assessment of the relation of these types of trait curiosity to creative performance, we also separately considered subsets of items that assessed Interest-type/Joyous Exploration versus Deprivation-type curiosity.

By including multiple ways of assaying curiosity, and multiple ways of assaying creativity, we aimed to capture, clarify, and consolidate our understanding of the curiosity-creativity connection. We hypothesized that each of the behaviorally-based measures of curiosity from the Curiosity Q&A Task (that is, active generation of novel topic-related questions and "gap-provoked" information foraging) would be positively correlated with creative ideation on the divergent thinking tests of creativity. Given that originality is a core aspect of creative or innovative thought, and is most directly related to novel ideational search, our hypotheses focused particularly on this dimension, but we also report assessments of fluency and flexibility. Additionally, we asked whether Interest-type/Joyous Exploration curiosity might differentially correlate with measures comprised of participants' (a) generation of novel questions, (b) foraging for topic-related information, and (c) their divergent creative thinking performance (e.g., originality of responses on the AUT and FIQ), whereas deprivation-type curiosity might be more closely related to measures comprised of participants' (a) generation of gap-provoked questions, (b) foraging for gap-related information, and (c) their performance on the two tasks (Remote Associates Task and Analogy Completion) that especially call upon convergent creative thinking. More specifically, our preregistered hypotheses (https://osf.io/9cbxs) were:

### **Hypothesis 1**

Curiosity as measured by the *novelty of topic-related questions* on the Q&A task will significantly positively correlate with *originality* on the divergent thinking tasks, including (a) originality on the Alternative Uses Task, and (b) originality on the Figural Interpretation Quest.

### **Hypothesis 2**

Curiosity as measured by *asking gap-related questions* and by *gap-provoked information foraging* during the Curiosity Q&A Task will significantly positively correlate with the proportion of *correct responses* on the (predominantly) convergent Remote Associates Task, and perhaps also the (predominantly) convergent Analogy Completion task.

## Methods

### Stimulus materials and scoring

A. **Assessments of curiosity**
1. **Question asking**
  In the Curiosity Q&A task, participants were presented with six brief factual statements about a variety of subjects, such as notable feats of mountain climbing, the origins of molecular gastronomy, and scientific findings relating to the use of hammocks. (See Supplementary Materials for the Curiosity Q&A task stimuli.) After each stimulus, participants were given the opportunity to ask—by typing into a text box—any questions that might naturally arise in relation to that stimulus.
  For example, participants might read the following factual stimulus: *"Although cochineal may seem like an unusual ingredient used during the manufacturing process of modern-day makeup, it has also been used to dye various other products."* After reading this statement, participants might type questions relating to the two implicit gaps in the statement, that is, "*What are cochineals?"* and "*What other products use cochineals as dye?"* Participants might also ask a diverse array of other topic-related questions, including questions that could be novel, atypical, or surprising such as *"How is it used in makeup?" "How long has cochineal been used?" "Is cochineal dangerous?" "Is it better than other dyes?" "Is it harmful to the environment?"* and *"Is it usually listed on the ingredient label?".*
  Participants' questions to each factual stimulus from the Curiosity Q&A task were separately and independently evaluated by two raters. The two raters first assessed whether the questions pertained to the implicit information gaps in the factual stimuli (gap-related Qs) or concerned other topic-related matters (topic-related Qs). Disagreements on the classification of responses as gap-related versus topic-related were resolved by discussion with a third independent rater. Then, for each factual stimulus, two raters evaluated the comparative novelty of participants' questions on a three-point scale (0 = not novel, 1 = somewhat novel, 2 = clearly novel). Interrater reliability for these assessments was excellent (*r* = 0.96); analyses are based on the average of the two raters' scores.
2. **Gap-provoked information foraging**
  After generating their questions to a given factual statement, participants were provided the opportunity to discover the missing information by clicking on one or both of 2 screen-displayed "buttons" that would reveal the answers to those gap-related questions. Additional buttons could reveal the answers to 7 other topic-related questions that they, or others, may have asked about the topic. These additional questions were developed in earlier pilot work with this paradigm. For the illustrative factual statement about cochineal, provided above, other topic-related questions that participants could choose to look at for answers included *"How is the dye made from cochineals?"* and *"Do humans ever respond poorly to cochineal dye?".* Clicking on a button would reveal the answer to the stated question. So, for instance, clicking on the gap-related button *"What are cochineals?"* revealed the answer, *"Cochineals are insects."* (See Supplementary Materials for the full set of Curiosity Q&A stimuli.) Participants could choose to look at the answers to any of the 9 questions associated with each factual statement (i.e., the 2 gap-related questions and the 7 topic-related questions), with no time limit, and, when they were ready, could advance to the next factual statement by clicking a "Next" button. The measure of gap-provoked information foraging was the number of times (out of the 2 opportunities per stimulus) that participants chose to "look" so as to view gap-related answers; the measure of topic-related information foraging was the number of times (out of 7 opportunities per stimulus) that participants chose to look to see topic-related information.
3. **Trait-based assessments of curiosity**
  To explore the relations between behaviorally-assessed curiosity and creativity and self-reported measures of curiosity and openness to experience, we administered: (a) the Five-Dimensional Curiosity Scale-Revised^47^, 24 items, newly added here, and (b) the Woo Openness to Experience measure^25^, 54-items; also administered in the pilot study. Given these are exploratory questions, and to avoid influencing behavioral assessments, the questionnaires of trait curiosity and openness to experience were administered after the key behavioral tasks.

B. **Assessments of creativity**

*Alternative Uses Task (AUT)* In the current implementation of this extensively used measure of divergent thinking^36,38,39^, participants were presented the names of three common objects (cardboard box, flashlight, wooden ruler), one at a time, and asked to indicate all of the unusual ways the object might be used. The typical or standard use of the object was indicated (e.g., for wooden ruler, "used for measuring lengths"). Participants responded without a time limit by typing their answers into a text box. Responses were evaluated by two independent condition-blind raters for fluency, flexibility, and originality according to lab-based scoring rubrics. Interrater reliability was strong for all three measures (*r* = 1.00 for fluency, *r* = 0.86 for flexibility, *r* = 0.86 for originality); analyses are based on the average of the two raters' scores.

**Figure 1:**
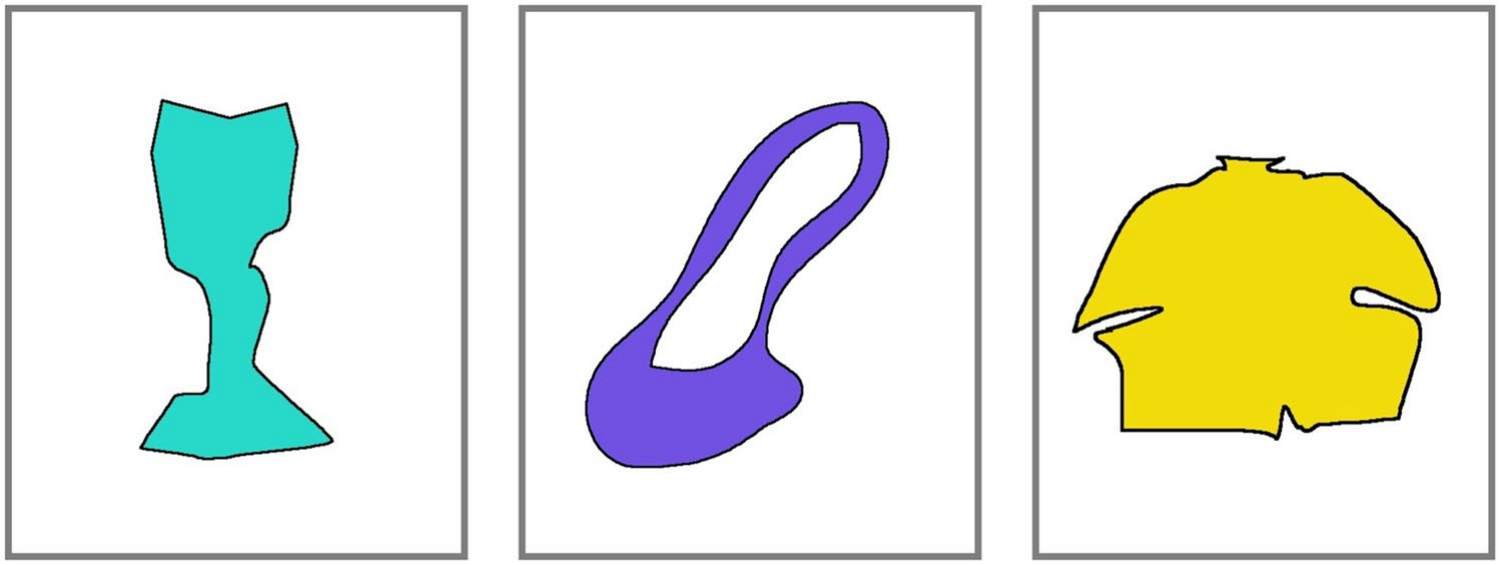
Illustrative items from the figural Interpretation Quest.

*Figural Interpretation Quest (FIQ)* Participants were shown three ambiguous colored line-drawn shapes, one at a time, and asked to indicate all of the various things the object might be. For example, a teal colored shape that fans out at both ends with a thinner middle may be interpreted as a wine glass, a vase, or a shovel. The stimuli were a selected subset of items originally developed to examine semantic contributions to episodic memory^52^, and adapted to provide a perceptually-prompted measure of divergent thinking^40–42^. Following the methods used in^42^, each item was shown for 40 s, but participants also could choose to advance to the next item if they chose to do so. Illustrative items from the FIQ are shown in Fig. 1.

Responses were scored by two independent condition-blind raters for fluency, category flexibility, and originality (3-point scale of 0, 1, or 2) according to lab-based scoring rubrics. Interrater reliability was excellent for all three measures (*r* = 1.00 for fluency, *r* = 0.92 for flexibility, *r* = 0.90 for originality); analyses are based on the average of the two raters' scores.

*Remote Associates Task (RAT)* In this task, participants were shown 20 problems, of a range of difficulty levels, drawn from published normative data^44^. For each problem, participants were shown three words (e.g., *safety, cushion, point*) and were tasked with identifying a fourth word that was related to, and could be meaningfully combined with, each of the three words. (In the given example, the correct response is *pin: safety pin, pin cushion, pinpoint*). Participants were given 4 min for this task.

*Analogy Completion Task* This task was not given in the pilot study but was previously administered in another recent study in our lab with a similar population. In the task, participants are shown an incomplete analogy and are asked to provide the missing term (e.g., *bear:cave :: bird: ___*). The items were selected from various published sources (e.g.^45^) and instructions on what makes a valid analogy were provided. Participants were given 4 min for this task. Participants' written analogy completion responses were assessed using the scoring rubric developed for the recent lab study. Some of the analogies were semantically-near comparisons (e.g., *kitten:cat::___:dog*) whereas others spanned different domains and were semantically distant (e.g., *tile:mop::tooth:___*). As noted in our Preregistration (https://osf.io/9cbxs), analogy scores were considered separately for the easier semantically-near analogies (10 items) versus semantically-distant analogies (18 items), with analyses focused on the semantically-distant items. Semantically-near items served as an attentional control for this online study; specifically, to be included, participants must have correctly answered 5 or more of the 10 semantically-near (easy) analogy completion items (in prior work, the average score for these items was 0.95).

*Pilot study findings and power analysis* The pilot study was conducted over two sessions, with the first session conducted individually and in-person, and the second session, approximately two days later, performed online via Qualtrics. A total 67 undergraduate participants (51 female, 16 male, *M* age = 19.68, *SD* = 2.06, *M* years education = 13.29, *SD* = 1.29) completed both days, including the Curiosity Q&A task on Day 1 and assessments of creative performance on Day 2. We based our power analyses on the primary research questions, and the effect sizes observed in this pilot study involving a similar population and behavioral measures. Specifically, for Hypothesis (1) Curiosity as measured by the novelty of (topic related) questions on the Q&A task will be positively correlated with originality on the divergent thinking tasks, the pilot study correlations were Novel Qs (all) with AUT Originality, *r* = 0.43; Novel Qs (all) with FIQ Originality, *r* = 0.48. For Hypothesis (2) Curiosity as measured by gap-related information foraging will be positively correlated with the proportion of correct responses on the Remote Associates Task, the pilot study correlations were, RAT Total, *r* = 0.50; RAT Medium Difficulty, *r* = 0.47, RAT High Difficulty, *r* = 0.41. Assuming, conservatively, that the effect sizes may be somewhat smaller, for *r* = 0.30, we need N = 112 to achieve power of 0.90 (for a nondirectional bias-corrected test).

### Experimental design and procedure

The study was completed entirely online (via Qualtrics). It employed a within-subjects experimental design, with all tasks and measures administered to all participants. There was one between-subjects factor of task order, which manipulated the order of the three main behavioral measures, including the Curiosity Q&A task, the Divergent thinking tasks, and the Convergent thinking tasks. There were an approximately equal number of participants in each of the three task orders of administration: Order A (Informed Consent, Q&A, Divergent, Convergent, Questionnaires, *n* = 61); Order B (Informed Consent, Divergent, Q&A, Convergent, Questionnaires, *n* = 56); Order C (Informed Consent, Convergent, Q&A, Divergent, Questionnaires, *n* = 62).

All experimental protocols were approved by the University of Minnesota Institutional Review Board (IRB) and all methods were carried out in accordance with relevant guidelines and regulations. Informed consent was obtained from all participants.

### Participants

A total of 189 participants completed the study; of these 10 participants were excluded from data analyses because they did not meet the study inclusion criteria (age range, native English speaking, *n* = 3), they failed the attention check (*n* = 1), *and/or* they correctly answered fewer than half of the control (easy) analogies (*n* = 8). Analyses were thus performed on data from 179 participants; in a few cases, data were missing for a single task or questionnaire; in these cases the corresponding sample size is reported.

Participants (154 female, 25 male) were, on average, 19.65 (*SD* = 1.74) years of age, and reported an average of 13.63 (*SD* = 1.33) years of formal education. Participants self-reported as being in generally good health (*M* = 5.70 on a 7-point Likert scale, *SD* = 1.09), and overall well-being (*M* = 5.05 on a 7-point Likert scale, *SD* = 1.21), and in a moderately positive overall mood (*M* = 6.02 on a 9-point Likert scale, *SD* = 1.53).

## Results

### Descriptive statistics

Table 1 presents the descriptive statistics for the Curiosity Q&A Task and the creative ideation measures. The final column of Table 1 also provides the inter-rater reliability (Pearson's *r*) for the rater-based assessments of the Curiosity Q&A Task measures and the divergent thinking tasks.

**Table 1:** Descriptive statistics for the Curiosity Q&A task and creative ideation measures.

Task and measure	Mean	SD	95% CI	Inter-rater reliability
**Curiosity Q&A Task**				
***Question Asking***				
Gap Questions	0.92	0.57	[0.83, 1.00]	0.99
Topic-Related Questions	1.64	1.80	[1.37, 1.91]	0.99
Novelty of Questions	0.68	1.16	[0.51, 0.85]	0.96
***Information Foraging***				
Looks at Gap Information	1.25	0.65	[1.15, 1.34]	–
Looks at Topic-Related Information	3.35	2.39	[3.00, 3.71]	–
**Divergent Thinking Tasks**				
***Figural Interpretation Quest (FIQ)***				
FIQ Fluency (*n* = 178)	4.14	1.41	[3.93, 4.35]	1.00
FIQ Flexibility (*n* = 178)	2.93	0.89	[2.80, 3.06]	0.92
FIQ Originality (*n* = 178)	2.43	1.40	[2.23, 2.64]	0.90
***Alternative Uses Task (AUT)***				
AUT Fluency (*n* = 177)	4.35	2.08	[4.04, 4.66]	1.00
AUT Flexibility (*n* = 177)	3.85	1.65	[3.60, 4.09]	0.86
AUT Originality (*n* = 177)	2.01	1.73	[1.75, 2.27]	0.86
**Convergent Thinking Tasks**				
***Remote Associates Task (RAT)***				
RAT Proportion Correct	0.33	0.18	[0.30, 0.36]	–
***Analogy Completion Task***				
Analogy Proportion Correct (All)	0.72	0.19	[0.70, 0.75]	–
Analogy Proportion Correct (Near)	0.86	0.15	[0.84, 0.88]	
Analogy Proportion Correct (Distant)	0.65	0.23	[0.61, 0.68]	–

Means for the Q&A task, FIQ, and AUT are per stimulus (six factual stimuli for the Q&A task, three FIQ stimuli, and three AUT stimuli); novelty and originality scores are the summed novelty or originality per stimulus. The maximum score per stimulus for Gap Questions and for Looks at Gap Information was 2; the maximum score per stimulus for Looks at Topic-Related Information was 7; there was no upper limit on the number of Topic-Related Questions that participants could choose to ask.

### Question asking and creative performance

Table 2 presents the Pearson correlations between the Curiosity Q&A task measures of question-asking and the creative ideation measures. From Table 2 it can be seen that, as hypothesized, Originality of responses on the AUT significantly and positively correlated with the Novelty of questions that participants asked on the Curiosity Q&A task (*r* = 0.35, *p* < 0.001); Originality of responses on the FIQ also positively correlated with Novelty of questions, but not significantly (*r* = 0.13, *p* = 0.093). Furthermore, as hypothesized, the frequency of asking Gap-related questions on the Curiosity Q&A task significantly positively correlated with the proportion of correct answers provided to the predominantly convergent thinking tasks, including both the RAT (*r* = 0.20, *p* = 0.007) and the Analogy Completion task (*r* = 0.21, *p* = 0.005).

**Table 2:** Correlation of curiosity Q&A task question-asking measures with creative ideation measures.

Creative ideation measure	Correlation with novel Qs [95% CI]	Correlation with topic-related Qs [95% CI]	Correlation with gap-related Qs [95% CI]
**Divergent thinking tasks**			
AUT Fluency	*r* = 0.28, *p* < 0.001*** [0.14, 0.41]	*r* = 0.34, *p* < 0.001*** [0.21, 0.47]	*r* = 0.21, *p* = 0.006** [0.06, 0.34]
AUT Flexibility	*r* = 0.22, *p* = 0.003** [0.08, 0.36]	*r* = 0.24, *p* = 0.001** [0.10, 0.37]	*r* = 0.12, *p* = 0.12 [− 0.03, 0.26]
AUT Originality	*r* = 0.35, *p* < 0.001*** **[0.22, 0.48]**	*r* = 0.34, *p* < 0.001 [0.20, 0.46]	*r* = 0.08, *p* = 0.28 [− 0.07, 0.23]
FIQ Fluency	*r* = 0.11, *p* = 0.13 [− 0.04, 0.26]	*r* = 0.16, *p* = 0.031* [0.02, 0.30]	*r* = 0.13, *p* = 0.078^ [− 0.02, 0.27]
FIQ Flexibility	*r* = 0.10, *p* = 0.20 [− 0.05, 0.24]	*r* = 0.12, *p* = 0.106 [− 0.03, 0.26]	*r* = 0.05, *p* = 0.53 [− 0.10, 0.19]
FIQ Originality	***r* = 0.13, *p* = 0.093^** **[− 0.02, 0.27]**	*r* = 0.13, *p* = 0.081^ [− 0.02, 0.27]	*r* = 0.04, *p* = 0.57 [− 0.11, 0.19]
**Convergent thinking tasks**			
RAT Proportion Correct	*r* = 0.12, *p* = 0.12 [− 0.03, 0.26]	*r* = 0.17, *p* = 0.024* [0.02, 0.31]	*r* = 0.20, *p* = 0.007** **[0.06, 0.34]**
Analogy Completion Proportion Correct	*r* = 0.21, *p* = 0.006** [0.06, 0.34]	*r* = 0.25, *p* < 0.001*** [0.11, 0.38]	*r* = 0.21, *p* = 0.005** **[0.06, 0.34]**

***p < 0.001, **p < 0.01, *p < 0.05, ^^^*p* < 0.10. All *p*-values are two-tailed. AUT, Alternative Uses Task; FIQ, Figural Interpretation Quest; RAT, Remote Associates Task; Analogy Completion is for the semantically-distant analogies. Values in bold font correspond to the preregistered hypothesized correlations; other correlations are reported for comprehensiveness and to allow comparisons.

### Gap-related information foraging and creative performance

We next evaluated the curiosity-creativity connection from the perspective of our second main behavioral measure of curiosity, namely the extent to which participants autonomously sought out information that was implicitly missing from the factual statements, or "gap-related information foraging." Here we calculated the number of gap-related "looks" in the Curiosity Q&A Task. Given that there were 6 factual stimuli, and each factual stimulus had 2 gaps, there were a total of 12 opportunities for participants to seek out the gap-related information. On average, participants chose to look at the gap-related information for 7.48 of the 12 gap-related buttons (95% CI: 6.91, 8.05), or an average of 1.25 out of 2 opportunities per stimulus.

Table 3 presents the correlations between the proportion of gap-related information foraging, and broader topic-related foraging with the creative ideation measures. As can be seen from Table 3, as hypothesized, the frequency of specifically *gap-related* information foraging was associated with higher accuracy on the two convergent-thinking tasks, with both performance on the RAT (*r* = 0.18, *p* = 0.018) and the Analogy Completion task (*r* = 0.18, *p* = 0.018) modestly but significantly correlated with gap-related looks, and not with more diffuse or general topic-related information foraging. The divergent thinking measures generally showed little association with either gap-related or topic-related information foraging, with the exception that AUT fluency significantly correlated with gap-related looks.

**Table 3:** Correlation of curiosity Q&A task information foraging measures with creative ideation measures.

Creative ideation measure	Correlation with gap-related looks [95% CI]	Correlation with topic-related looks [95% CI]
**Divergent thinking tasks**		
AUT Fluency	*r* = 0.17, *p* = 0.022* [0.03, 0.31]	*r* = 0.10, *p* = 0.17 [− 0.05, 0.25]
AUT Flexibility	*r* = 0.12, *p* = 0.11 [− 0.03, 0.26]	*r* = 0.07, *p* = 0.36 [− 0.08, 0.21]
AUT Originality	*r* = 0.09, *p* = 0.21 [− 0.05, 0.24]	*r* = 0.05, *p* = 0.48 [− 0.10, 0.20]
FIQ Fluency	*r* = 0.09, *p* = 0.22 [− 0.06, 0.24]	*r* = 0.06, *p* = 0.43 [− 0.09, 0.21]
FIQ Flexibility	*r* = 0.06, *p* = 0.43 [− 0.09, 0.21]	*r* = 0.02, *p* = 0.75 [− 0.12, 0.17]
FIQ Originality	*r* = 0.06, *p* = 0.44 [− 0.09, 0.20]	*r* = 0.08, *p* = 0.28 [− 0.07, 0.23]
**Convergent thinking tasks**		
RAT Proportion Correct	*r* = 0.18, *p* = 0.018* **[0.03, 0.32]**	*r* = 0.07, *p* = 0.34 [− 0.08, 0.22]
Analogy Completion Proportion Correct	*r* = 0.18, *p* = 0.018* **[0.03, 0.32]**	*r* = 0.06, *p* = 0.42 [− 0.09, 0.21]

***p < 0.001, **p < 0.01, *p < 0.05, ^^^*p* < 0.10. All *p*-values are two-tailed. AUT, Alternative Uses Task; FIQ, Figural Interpretation Quest; RAT, Remote Associates Task; Analogy Completion is for the semantically-distant analogies. Values in bold font correspond to the preregistered hypothesized correlations; other correlations are reported for comprehensiveness and to allow comparisons.

### Trait-based self-reported curiosity and creative ideation measures

Table 4 provides the descriptive statistics for the Woo et al.^25^ Openness to Experience Questionnaire and the Five-Dimensional Curiosity Scale-Revised^47^. Correlations of the three trait-based curiosity measures (Woo Curiosity, Joyous Exploration, and Deprivation Sensitivity) with each of the creative ideation measures are provided in Supplementary Materials, Table S1. Additionally, given the well-established association between openness to experience and creative ideation^23,24^, to allow examination and comparisons of specifically the contribution of the curiosity component of Openness to creative ideation, Supplementary Materials, Table S2 gives the correlations between the Global measure of Openness (that does not separately consider curiosity but simply averages it together with all of the other facets into the overall score), and also the intermediate aspects of Intellect (which includes Efficiency, Ingenuity, and Curiosity) and Culture (which includes Aesthetics, Tolerance, and Depth) with each of the creative ideation measures.

**Table 4:** Descriptive statistics for the trait openness to experience and curiosity measures.

Questionnaire and subscale	Mean	SD	95% CI
**Woo Openness to Experience**			
Efficiency	3.04	0.72	[2.94, 3.15]
Ingenuity	3.36	0.68	[3.26, 3.46]
Curiosity	3.83	0.52	[3.75, 3.91]
*Intellect Aspect*	3.41	0.52	[3.33, 3.49]
Aesthetics	3.24	0.93	[3.10, 3.37]
Tolerance	3.99	0.48	[3.91, 4.06]
Depth	3.63	0.60	[3.55, 3.72]
*Culture Aspect*	3.62	0.56	[3.54, 3.70]
*Global Openness*	3.51	0.46	[3.45, 3.58]
**5-Dimensional Curiosity Scale-Revised**			
Joyous Exploration	5.18	0.98	[5.03, 5.32]
Deprivation Sensitivity	4.37	1.30	[4.18, 4.56]
Stress Tolerance (reversed)	3.77	1.20	[3.59, 3.94]
Thrill Seeking	3.76	1.16	[3.59, 3.94]
Overt Social	5.51	0.94	[5.37, 5.65]
Covert Social	5.37	1.15	[5.20, 5.54]

Items on the Openness to Experience questionnaire^25^ are on a 5-point scale (1 = strongly disagree, 5 = strongly agree). Items on the 5-Dimensional Curiosity Scale-Revised^47^ are on a 7-point scale (1 = does not describe me at all, 7 = completely describes me). For all trait measures, *N* = 178. Means for Openness to Experience are presented at the lowest level of all 6 facets, at the intermediate aspect levels of *Intellect* (combining Efficiency, Ingenuity, and Curiosity) and *Culture* (combining Aesthetics, Tolerance, and Depth), and also at the overall trait level of *Global Openness* (combining both aspects).

Next, to provide a more fine-grained probe of the relation between trait curiosity and creative performance, we separately considered the Interest-type/Joyous Exploration and Deprivation-type items in relation to comparatively divergent (AUT and FIQ) and the comparatively convergent (RAT and Analogy Completion) creative tasks, in conjunction with comparatively divergent curiosity measures (asking novel questions and foraging for topic-related answers) and comparatively convergent curiosity measures (asking gap-related questions and foraging for gap answers). These correlations are found in Table 5, with the upper panel (Panel A) focusing on intercorrelations with the predominantly divergent measures and the lower panel (Panel B) focusing on intercorrelations with the predominantly convergent measures.

**Table 5:** Intercorrelations of predominantly divergent versus predominantly convergent measures with Joyous Exploration, Deprivation Sensitivity, and Woo Trait Curiosity Measures.

Measure	1	2	3	4	5	6	7
**A. Predominantly divergent: asking novel questions and foraging for topic-related answers**							
1. Novel Questions	–						
2. Topic-Related Foraging	0.20**	–					
3. Deprivation Sensitivity	0.12	− 0.10	–				
4. Joyous Exploration	0.19**	0.08	0.58***	–			
5. Woo Curiosity	0.16*	0.06	0.51***	0.73***	–		
6. AUT Originality	0.35***	0.05	0.15*	0.17*	0.22**	–	
7. FIQ Originality	0.13^	0.08	− 0.02	0.08	0.11	0.42***	–
**B. Predominantly convergent: asking gap questions and foraging for gap answers**							
1. Gap Questions	–						
2. Gap-Related Foraging	0.50***	–					
3. Deprivation Sensitivity	− 0.02	− 0.01	–				
4. Joyous Exploration	0.04	0.10	0.58***	–			
5. Woo Curiosity	0.04	0.07	0.51***	0.73***	–		
6. RAT Correct	0.20**	0.18*	− 0.01	0.09	0.02	–	
7. Analogy Completion	0.21**	0.18*	0.04	0.18*	0.15*	0.43***	–

***p < .001, **p < .01, *p < .05, ^^^*p* < .10. All *p*-values are two-tailed.

From Table 5 it can be seen that the two predominantly *divergent* Curiosity Q&A measures of posing novel questions and foraging for topic-related information were significantly correlated with one another (*r* = 0.20), as were the two predominantly *convergent* Curiosity Q&A measures of posing the gap questions and gap-related foraging (*r* = 0.50). Additional noteworthy observations from Table 5 are the robust intercorrelations of the two divergent thinking task assessments of originality (AUT originality with FIQ originality, *r* = 0.42) and between performance on the two convergent thinking tasks (RAT and Analogy Completion, *r* = 0.43). There were also strong intercorrelations of the three self-report measures of curiosity, including with deprivation sensitivity (*r* = 0.58 and *r* = 0.51), with an especially strong correlation between the joyous exploration measure and the Woo Curiosity subscale (*r* = 0.73).

Finally, as an exploratory and broadly integrative overview analysis, we performed a principal components analysis on all of the behaviorally-assessed indices of curiosity and creative ideation. As shown in Table 6, this analysis yielded five components with eigenvalues greater than one, and inclusion of the five components, with no rotation, explained approximately 82% of the variance.

**Table 6:** Principal components analysis on all behaviorally-assessed indices of curiosity and creative ideation.

Component	Total	% of variance	Cumulative %		
1	4.46	34.29	34.29		
2	2.47	19.03	53.32		
3	1.43	11.00	64.30		
4	1.24	9.54	73.84		
5	1.06	8.15	81.97		

Measure	Component				
	1	2	3	4	5
Novel Questions	0.56	0.50	− 0.39	0.19	− 0.32
Gap-related Questions	0.45	0.59		0.20	− 0.16
Topic-related Questions	0.63	0.60	− 0.28	0.18	− 0.29
Gap-related Looks	0.42	0.65	0.42	− 0.36	0.16
Topic-related Looks	0.31	0.55	0.45	− 0.52	0.13
AUT Fluency	0.80	− 0.23	− 0.30	− 0.21	0.24
AUT Flexibility	0.66	− 0.22	− 0.36	− 0.23	0.37
AUT Originality	0.74	− 0.24	− 0.40	− 0.19	0.18
FIQ Fluency	0.68	− 0.44	0.36		− 0.32
FIQ Flexibility	0.67	− 0.50	0.27		− 0.24
FIQ Originality	0.63	− 0.43	0.31		− 0.29
RAT Proportion Correct	0.38		0.31	0.54	0.45
Analogy Completion	0.45		0.21	0.56	0.35

For clarity, values less than 0.10 are not reported. Analogy completion is for the semantically-distant analogies.

From Table 6, it can be seen that all of the curiosity and creative ideation measures loaded positively on the first component, and together explained about one-third of the variance. This suggests that there is indeed a common latent construct that fuels both curiosity and creative ideation. The second component, however, bifurcated the indices of these two constructs with strong positive weightings of all of the Curiosity Q&A task measures, including both question-asking and information foraging, together with negative weightings from the two divergent-thinking tasks. This thus suggests that—despite their commonality—curiosity and divergent creative ideation also differ from one another/partially rely on different mechanisms. The third component mainly involved positive weightings from tasks that may be grouped in that they provide relatively greater perceptual or external-environmental scaffolding for idea search, including the information-foraging measures, the perceptually-prompted FIQ, and the two convergent-thinking tasks, accompanied by negative weightings for tasks demanding more autonomously-guided internal search (question generation and AUT responding). The fourth and fifth components also both have positive weightings from the two convergent-thinking tasks but further differentiate between question-asking and information-foraging and also differentiate between the two divergent-thinking tasks.

## Discussion

The primary aim of this preregistered study was to examine the association between curiosity and creative ideation when (a) both curiosity and creative ideation are assessed with behaviorally-observed outcomes (rather than only self-report) and (b) both curiosity and creativity are assessed using behavioral measures that assess core aspects of each construct. For curiosity the two core aspects involve: (1) internally generated exploration and identification of what an individual does not know or desires to know, here operationally assessed through the formulation and posing of questions ("question asking") regarding experimentally presented factual information; (2) externally supported exploratory information seeking, here operationally assessed through an individual's choosing to look at the answers to visually provided questions ("information foraging"). For creativity the two core aspects involve: (1) divergent thinking, the generation of multiple, varied, and original ideas in response to an open-ended problem, for which a large number of possible responses are acceptable and may be deemed correct; (2) convergent thinking, the generation of the single best (or correct) answer to a clearly defined problem or question.

We hypothesized that curiosity, as measured by the *novelty of topic-related questions* on the Q&A task, would significantly positively correlate with *originality* on the divergent thinking tasks, including (a) originality on the Alternative Uses Task^36,39^ and (b) originality on the Figural Interpretation Quest^40–42^. We further hypothesized that curiosity as measured by *asking gap-related questions* and by *gap-provoked information foraging* during the Curiosity Q&A Task would significantly positively correlate with the proportion of *correct responses* on the (predominantly) convergent Remote Associates Task, and perhaps also the predominantly convergent Analogy Completion task.

The results partially supported the first hypothesis and fully supported the second hypothesis. Our primary behavioral curiosity measure of novel question-asking was positively associated with the independently-assessed behavioral measure of originality on the Alternative Uses Task (AUT). Additionally, we found evidence that our second behavioral curiosity measure of gap-provoked information foraging was especially linked to performance on the RAT and the Analogy Completion task, which are predominantly convergent-thinking measures of creativity. These outcomes are among the first empirical demonstrations of a strong positive curiosity-creativity connection under conditions where *both* of those constructs are behaviorally and independently assessed in performance-based tasks, rather than one or both constructs being indexed by self-report.

The comparatively weaker correlation between our curiosity measure of novel question-asking and originality on the FIQ task was unanticipated, and differed from our finding in the pilot study, which showed a significant positive correlation between FIQ originality and novel questions. It is notable that, in the current study, this weaker correlation was nonetheless accompanied by other indications that (as expected) the AUT and FIQ were both successful prompts for divergent ideation; for instance, there was a strong correlation between originality of responses on the two divergent-thinking measures (AUT originality correlated with FIQ originality, *r* = 0.42, *p* < 0.001). One possible explanation focuses on the differences in the material for the two divergent-thinking tasks, in particular, that there is a closer match between the verbally-based Curiosity Q&A task and the verbally-presented AUT items than between the Q&A task stimuli and the perceptually-based FIQ items. Another possible interpretation is that the association between behaviorally-assessed curiosity and original creative ideation may emerge more strongly when the divergent ideation task is untimed (like the AUT in the current study) rather than timed (like the FIQ in the current study). Indeed, the stronger association between FIQ Originality and Novel Questions that was observed in the pilot study did involve an untimed administration of the FIQ task and the average fluency, flexibility, and originality for the FIQ were numerically higher in the pilot study than in the current study.

Our more fine-grained probe of the relation between trait curiosity and creative performance, separately considering the Interest-type/Joyous Exploration and Deprivation-type items in relation to the comparatively divergent (AUT and FIQ) versus comparatively convergent (RAT and Analogy Completion) creative tasks, yielded little support for a uniquely predictive role of Deprivation-type curiosity for convergent creativity. Deprivation-type curiosity did not correlate either with the posing of gap questions or with foraging for gap answers, and was largely uncorrelated with performance on both the RAT and the Analogy Completion tasks. Similar outcomes were observed in our pilot study. It is possible that deprivation-type curiosity is more closely tied to an individual's specific individuated goals or longer-term cognitive-motivational concerns, that are not well-typified by brief and incidental exposure to experimental materials. For instance, the types of problems that might lead one to strongly endorse the item, "I can spend hours on a single problem because I just can't rest without knowing the answer" may be quite different from the knowledge gaps implicitly hinted at in the Curiosity Q&A factual statements.

In contrast, there was stronger support for associations between especially Interest-type/Joyous Exploration curiosity and more divergent forms of inquiry (posing novel questions) and originality of ideation responses for the AUT. In general, Interest-type/Joyous-Exploration curiosity showed intercorrelations similar to those of the Woo curiosity subscale^25^, with which it was highly correlated. The majority of the 9 items on the Woo curiosity subscale do appear to be oriented to Interest-type curiosity (e.g., "I try to learn something new every day"; "I love to do experiments and see the results"). Consistent with this, a recent psychometric network analysis of several openness to experience measures^53^ found that Woo's curiosity subscale was associated with emergent facets of intellectual curiosity, nontraditionalism, diversity, and intellectual interests.

Despite the new probative evidence for a curiosity-creativity connection that this study has provided—particularly demonstrating a strong positive association of behaviorally-based assessments of curiosity (assayed by question asking and information foraging) with independently behaviorally-based assessments of creativity (assayed by creative performance on the AUT, RAT, and Analogy Completion)—some limitations should be noted. That all of the participants were college-aged students in the United States, and predominantly female, may constrain generalizability. Additionally, the study was completed entirely online. On the one hand, several quality-control mechanisms such as attention-check items were in place, and it is possible that online compared with in-lab assessments may allow more comfortable environments that, in turn, allow for more natural responding—considerations arguably important for creative ideation. On the other hand, the particular contexts under which participants complete an online research study vary and are uncontrolled. Furthermore, although the inclusion of several ways of behaviorally assessing both curiosity (indicated by the autonomous generation of questions and the frequency of searching for information that might be missing from one's knowledge network) and creative ideation (indicated by independent assessments of both divergent and convergent ideation) is a noteworthy strength, both curiosity and creativity are complex and multi-faceted constructs. While our exploratory principal component analysis on all of the behaviorally-based assessments of these constructs demonstrated clear commonalities between them (explaining 34% of the variance), it also underscored differences. One such difference is the extent to which a task is externally environmentally-supported (e.g., information foraging) compared with a task that requires more autonomously-guided, often effortful^54^ internal cognitive search (e.g., question generation). For example, question asking loaded strongly on the first two components, but information foraging additionally loaded on the third component. We also saw that performance on the convergent measures (RAT and Analogy Completion) loaded on four of the five components––an observation congruent with the position that most cognitive tasks are not "process pure." For instance, although the Analogy Completion task is largely convergent in nature, it also calls upon divergent ideation^55^.

Another limitation of the current research is that, despite newly empirically documenting the correlation of behaviorally-assessed curiosity with behaviorally-assessed creativity, the directionality of the relation cannot be determined from the present findings. Does curiosity fuel creativity, does creativity ignite curiosity, or are both in some measure true? One hint at the directionality of these associations may come from an examination of the between-subject effects of task order in the current study, where the Curiosity Q&A task was sometimes administered as the first task, sometimes followed the divergent-thinking tasks, and sometimes followed the convergent-thinking tasks. The generation of novel questions, and also of topic-related questions, during the Curiosity Q&A task was especially elevated when the Q&A task *followed* the divergent-thinking tasks (Order B) compared both to when the Q&A task was the first cognitive task administered (Order A), and to when the Q&A task was given after the convergent-thinking tasks (Order C) [see Supplementary Materials, Table S3 and Table S4]. This may suggest that, at least on a shorter time-scale, engaging in creative activities that recruit divergent thinking processes may bolster subsequent curiosity. Additional research on such across-task cognitive-procedural priming effects is warranted (see also^56^).

From a pedagogical, organizational, and social-community perspective, the evidence provided here that there is a strong behavioral connection between curiosity and creative ideation underscores the importance of continuing to develop interventions that foster *both* creative thinking and active autonomous inquiry. The striking similarities between process-based theoretical approaches to creativity and active self-directed learning, noted earlier^31,32^, are relevant here. Both innovative thinking and inquiry-based learning entail flexibly exploring information from different sources and perspectives, generating new ideas, and combining or reconfiguring what one knows. Both likewise hinge upon cognitive-motivational factors such as a sense of intellectual engagement and autonomy that an individual's (or team's) working and learning environment can support—or squelch^57–59^. Promoting—and individually and collectively modelling—a welcoming receptivity to experimentation^60^, to divergently asking questions^1,61^, and openly seeking feedback^62^ are all promising places to start, especially if combined with a positive, learning attitude toward uncertainty, ambiguity, and the occurrence of failures^31,60^. Neither engaging in acts of curiosity, nor attempts at creative flexibility, are guaranteed success, and becoming comfortable with uncertainty and ambiguity is integral to the forging of new and valuable ways of doing and thinking^63–65^.

A recent review titled, "cultivating an understanding of curiosity as a seed for creativity" concluded that "our understanding of the curiosity-creativity relationship is limited" and that research on this topic is "hampered by methodological limitations, particularly the scarcity of state and behavioral measures, as well as experimental manipulations evoking states of curiosity" (p. 80)^7^. The work presented here takes a step in the needed direction. Many additional steps are necessary, if we are to fully "capture, clarify, and consolidate the curiosity-creativity connection." But we'll get there—with sufficient (shared, collaborative) creative thinking, plus a healthy dose of curiosity.

## Supplementary Information

Supplementary Information.

## Data Availability

The datasets generated during and analysed during the current study are available in Harvard Dataverse: Koutstaal, Wilma, 2022, "The Creativity Curiosity Connection—Preregistered Replication Study", https://doi.org/10.7910/DVN/R6WTNN, Harvard Dataverse, V1.
