# Supplementary material for: Capturing, clarifying, and consolidating the curiosity-creativity connection
Source: Sci Rep. 2022 Sep 12;12:15300. doi: 10.1038/s41598-022-19694-4 (PMC9468176; doi:10.1038/s41598-022-19694-4)

**Supplementary Materials for**

**"Capturing, Clarifying, and Consolidating the Curiosity-Creativity Connection"**

Wilma Koutstaal, Kara Kedrick, & Joshua Gonzalez-Brito

**1. Curiosity Q&A Task Stimuli**

*Provided below is the full set of stimuli presented in the Curiosity Q&A task in the current study. There were six factual stimuli (plus a practice stimulus), listed here as Factual Stimulus 01 through Factual Stimulus 06. The two "gap-related questions" for each stimulus, and the associated "answers" at which participants might look during the information foraging phase, are provided directly after each factual statement, followed by the seven "topic-related" questions, and the associated "answers" at which participants might also choose to look. The identifiers [Q1] through [Q9] indicate where, in the list of nine questions per stimulus, the gap-related and topic-related questions were presented.*

**Factual Stimulus 01: Favorite Number**

Alex Bellos was prompted by his public speaking appearances to create an online survey asking people to report their favorite number. After collecting 30,000 responses, there was a clear favorite.

[Q8]

**Gap 1:**

Q: What is the most popular favorite number?

A: The most popular favorite number was the number 7.

[Q1]

**Gap 2:**

Q: Why did Alex Bellos’ public speaking appearances prompt him to create an online survey?

A: During his talks on mathematics, Alex Bellos was often asked what was his favorite number. Through subsequent audience exchanges, he realized that people tend to have an emotional connection to their own favorite number. He created the survey to better understand specific characteristics of that emotional connection.

**Topic-Related Questions:**

[Q2]

Q: What was the smallest number that wasn’t picked as someone’s favorite?

A: The smallest number that wasn’t picked was 110, which is an example of the fact that round numbers were seldom picked as favorite numbers.

[Q3]

Q: Who is Alex Bellos?

A: Alex Bellos is a popular writer and public speaker, who frequently writes and talks about mathematics. One of his best-known books is called *Alex’s Adventures in Numberland*, which examines the use of math throughout the world.

[Q4]

Q: What was the most common reason for initially choosing a favorite number?

A: The most common reason was that it was the day on which the person was born, but participants were less likely to choose their birthday as their favorite number if it ended in either 0 or 5. Bellos hypothesized that the reason for this exception is that numbers ending in 0 or 5 are commonly used as approximations and, therefore, don’t feel unique or special.

[Q5]

Q: Did people like even numbers more than odd numbers?

A: There wasn’t a significant difference between how many even versus odd numbers were selected as one’s favorite.

[Q6]

Q: What does Alex Bellos discuss during public speaking appearances?

A: Alex Bellos has appeared as the keynote speaker at schools and business conferences where he has discussed such topics as how mathematics can shape our view of the world, and the culture and history of Brazil – where he lived for a number of years.

[Q7]

Q: Were smaller numbers more often chosen as a favorite number?

A: Almost half of the numbers given as favorites were between 1 and 10.

[Q9]

Q: How many different favorite numbers were chosen?

A: Out of 30,025 submissions, 1,123 different numbers were selected.

**Factual Stimulus 02: Hammocks**

Although today hammocks are mostly associated with relaxation, they have different historical origins and recent scientific findings point to other reasons for their use.

[Q9]

**Gap 1:**

Q: What were hammocks’ historical uses?

A: Originally made of bark or fibers, they were used to provide safety from animal bites, insect stings, or disease transmission.

[Q5]

**Gap 2:**

Q: What other uses are indicated by hammocks’ scientific findings?

A: Research has found that people fall asleep faster in a hammock-like bed. The rocking induces slower oscillations of brain activity, which are associated with deep sleep and memory consolidation. Traditions of rocking to promote sleep may have evolved for this reason.

**Topic-Related Questions:**

[Q1]

Q: Where did hammocks originate?

A: Hammocks, dating back to pre-Columbian antiquity, were found from southern Brazil to central Mexico.

[Q2]

Q: How did Americans incorporate hammocks into their lifestyle?

A: In the late 19^th^ century, hammocks were used both by frontier farmers, for sleeping, and wealthy Americans, for relaxation.

[Q3]

Q: Who introduced hammocks to the Europeans?

A: Christopher Columbus and his men were the first Europeans to be introduced to hammocks by the Taino people in the Bahamas. They ended up bringing hammocks back to Spain.

[Q4]

Q: Did the army ever use hammocks?

A: An American physician named William Gorgas used hammocks in attempts to eradicate yellow fever while constructing the Panama Canal. He used hammocks as opposed to traditional beds because they could be covered in mosquito netting and distanced sleepers from the ground-dwelling insects.

[Q6]

Q: When did hammocks become primarily used for relaxation?

A: Hammocks were first used for relaxation by wealthy American families at the end of the 19th century.

[Q7]

Q: How were hammocks originally used by the Europeans?

A: Hammocks were originally adapted for use in European navy sailing ships, where there was limited space and where the hammock could move in synchrony with the motion of the ship. A hammock is more comfortable than a ship's bunk because the sleeper always stays well balanced.

[Q8]

Q: When and where were hammocks first mass produced?

A: Hammocks were first mass produced in Pawleys Island, South Carolina, in 1889.

**Factual Stimulus 03: Cochineal**

Although cochineal may seem like an unusual ingredient used during the manufacturing process of modern-day makeup, it has also been used to dye various other products.

[Q1]

**Gap 1:**

Q: What are cochineals?

A: Cochineals are insects.

[Q3]

**Gap 2:**

Q: What other products use cochineals as dye?

A: They have been used for everything from dyeing cotton to natural food coloring. Some products that they have been used in include: Jell-O, sausages, pies, shrimp, and jams.

**Topic-Related Questions:**

[Q2]

Q: What component of the cochineal is used to dye makeup?

A: The component is carmine taken from the gut of the insect. Its biological use is to protect the bug from predators.

[Q4]

Q: What do cochineals look like?

A: The bugs are small, flat, and oval-shaped. They are black and white in color.

[Q5]

Q: What is the color of the dye and which makeup products use cochineal dye?

A: Cochineal dye is a bright red pigment used in red and purple makeup such as lipstick and eyeshadow.

[Q6]

Q: How is the dye made from cochineals?

A: The dye is made by drying the insects and grinding them into a powder. The red pigment emerges when the powder is combined with water.

[Q7]

Q: Where are cochineals located and harvested?

A: Cochineals can be found in desert locations throughout the world. They are harvested – for the purpose of making dye – in Peru and the Canary Islands.

[Q8]

Q: What makes cochineal dye effective as a pigment?

A: It is one of the most light-stable, heat-stable, and oxidation-resistant of all the natural organic colorants. It is even more stable than many synthetic pigments.

[Q9]

Q: Do humans ever respond poorly to cochineal dye?

A: In 2009, the Food and Drug Administration found that people were having allergic reactions to cochineal dye, causing many manufacturers to drop cochineal from their ingredients list. For example, in 2012 Starbucks stopped using it in their Strawberry and Crème Frappuccinos.

**Factual Stimulus 04: Molecular Gastronomy**

Unique circumstances led to the birth of Molecular Gastronomy. This cooking method uses physics and chemistry to transform food, leading to techniques like spherification.

[Q4]

**Gap 1:**

Q: What is spherification?

A: Spherification is the process of turning liquid into gelified spheres that burst in one's mouth.

[Q6]

**Gap 2:**

Q: What unique circumstances led to the birth of Molecular Gastronomy?

A: An English culinary teacher named Elizabeth Thomas, who was connected to both the cooking community by profession and the physics community through her husband, created a cooking workshop that brought people together from both communities. This collaboration led to Molecular Gastronomy.

**Topic-Related Questions:**

[Q1]

Q: What processes lead to spherification?

A: There are two common processes: basic spherification and reverse spherification. Basic spherification involves adding sodium alginate to a liquid and then submerging it in a bath of calcium, whereas reverse spherification involves adding calcium to a liquid and then submerging it in a bath of sodium alginate.

[Q2]

Q: How does Molecular Gastronomy differ from traditional food science?

A: Historically, food science focused on improving the industrial production of food and identifying nutritional value. Molecular Gastronomy introduced innovative scientific methods to explore and expand the full sensory experience of food.

[Q3]

Q: What are other techniques used in Molecular Gastronomy?

A: Other techniques include: gelification, which is the process of turning liquids into gelatinous forms; emulsification, which is the process of creating foams; and flash frozen, which freezes food almost immediately by using liquid nitrogen.

[Q5]

Q: How has Molecular Gastronomy been received by the culinary community?

A: The incorporation of these techniques helped non-traditional chefs, such as Ferran Adria, create restaurants that rank amongst the greatest in the world. Traditionalists and restaurant critics tend to disparage this cooking style, arguing that food preparation should not be treated as a laboratory experiment and emphasizing the importance of natural ingredients.

[Q7]

Q: Where is Molecular Gastronomy commonly practiced?

A: The culinary world has embarked on Molecular Gastronomy-related research and educational initiatives in countries such as Denmark, Italy, Spain, France, and the United States. Due to the advanced scientific techniques involved in Molecular Gastronomy, it is more commonly used by chefs than by home cooks.

[Q8]

Q: Why is this cooking method called Molecular Gastronomy?

A: The term “Gastronomy” was chosen because it is commonly defined as “the intelligent knowledge of whatever concerns man’s nourishment.” The adjective “Molecular” was added to emphasize that this form of cooking relates to branches of science involving physics, chemistry, and biology.

[Q9]

Q: Does the nutritional value change after using Molecular Gastronomy to transform food?

A: This cooking style primarily changes the structure of food rather than its nutritional value.

**Factual Stimulus 05: Rock Climbing**

In 2012, Alex Honnold, a professional rock climber, encountered a complication on the first face of Yosemite’s famous Triple Crown, while doing a climb that had never been done before.

[Q2]

**Gap 1:**

Q: What complication occurred when Alex Honnold was climbing the first face of the Triple Crown?

A: As he was climbing the first face, he found himself swarmed by dozens of silverfish, which are silver, inch-long, wingless insects with wispy antennae. They found their way into his mouth, ears, and clothes.

[Q4]

**Gap 2:**

Q: What did Alex Honnold do that had never been done before?

A: He successfully climbed the Triple Crown, the three biggest rock faces in Yosemite, in succession, alone, and in less than 24 hours.

**Topic-Related Questions:**

[Q1]

Q: Which climbing technique did Alex Honnold use?

A: He used the free-soloing technique, where one climbs alone mostly without gear or rope.

[Q3]

Q: What are the names of the three rock faces of the Triple Crown?

A: The three rock faces are the walls of the south face of Mount Watkins (first face), Free Rider of El Capitan (second face), and the Regular Northwest Face of Half Dome (third face).

[Q5]

Q: How long did it take Alex Honnold to finish the climb?

A: He finished the climb in a record-breaking 18 hours and 59 minutes, a climb which normally takes most people several days to complete.

[Q6]

Q: How do most people climb the Triple Crown and how many people have succeeded?

A: Most climbers use equipment, such as hooks, anchors, and ropes, and typically climb with at least one other person over the course of several days. Since the trail’s creation in 1994, approximately 260 people have climbed the Triple Crown.

[Q7]

Q: What is Yosemite?

A: Yosemite is a national park located in California’s Sierra Nevada mountains.

[Q8]

Q: How many feet was the climb?

A: Alex Honnold climbed approximately 7,000 vertical feet.

[Q9]

Q: Had Alex Honnold climbed the Triple Crown beforehand?

A: Yes, Alex Honnold and Tommy Caldwell, another professional climber, climbed the Triple Crown together in 2012 with a record-breaking time of 21 hours and 15 minutes.

**Factual Stimulus 6: Capybaras**

Capybaras are the heaviest living rodent on earth, eating about 5 pounds of food a day. They have specific body features that allow them to live in semi-aquatic habitats in South America.

[Q3]

**Gap 1:**

Q: What body features allow the capybara to live in a semi-aquatic environment?

A: They have webbing between their fingers and toes, which helps them paddle, and eyes and ears positioned high on their heads, which allows them to hide well underwater.

[Q6]

**Gap 2:**

Q: How much do capybaras tend to weigh?

A: Capybaras often weigh around 175 pounds.

**Topic-Related Questions:**

[Q1]

Q: How much do humans eat in a day?

A: Research shows that most humans eat between three to five pounds of food per day.

[Q2]

Q: What animals prey on capybaras?

A: When the capybaras are on land, their predators include pumas, jaguars, and anacondas. Capybaras flee to water for safety, where there are few to no predators that hunt the adult population. On the other hand, young capybaras may be attacked by piranhas while in the water.

[Q4]

Q: What is a semi-aquatic environment and what types do capybaras inhabit?

A: A semi-aquatic environment is an environment where a living creature either lives in or close to water or carries out part of its life cycle in water. Capybaras live along river banks, ponds, and lakes.

[Q5]

Q: What do capybaras eat?

A:  Capybaras enjoy aquatic reeds and grasses. They also eat crops, such as wheat, melons, and squash.

[Q7]

Q: What areas in South America are inhabited by capybaras?

A: They are found in many countries, including Venezuela, Argentina, and Brazil.

[Q8]

Q: Do humans eat capybaras?

A: Yes, capybaras are hunted by humans in Venezuela.

[Q9]

Q: What do capybaras look like?

A: They resemble giant guinea pigs and range from 3.2 to 4.2 feet long.

**2. Divergent Creativity Task Instructions**

**Welcome to the Alternative Uses Task!**

In this task, you will be asked to consider a common object. The object has a common use, which will be stated. You are to list other possible uses for this object or parts of the object. List as many different uses as possible. Place each idea on a separate line.

Please try to be **creative and appropriate** in your responses.

Example: Given: a NEWSPAPER (used for reading). You might think of the following other uses for a newspaper:
1. Start a fire
2. Serve fish and chips
3. Swat flies

Please **try to provide about 5 or more responses for each object**. Make sure to fully understand the instructions before clicking Next. You will not be able to return to this page.

**-----**

**Welcome to the Figural Interpretation Quest!**

In this part, we will ask you to tell us: **What could each object be?** Give as many different interpretations as you can.

You will see **3 objects**, shown one at a time. For each object, you will have **40 seconds** to provide your ideas.  The color of each object **does not** matter. Type each interpretation on a separate line.

Here is an example of the type of objects you will be shown:

              
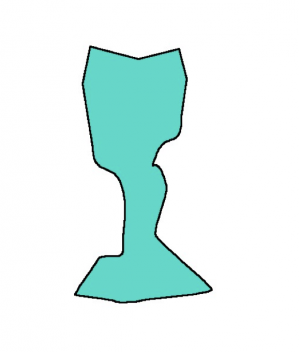


Before we go on, please practice giving two different interpretations of the example object in the space below.  (For the practice, we only ask that you give two responses.)

Get ready to be creative!  You will have 40 seconds per object.  Please make sure that you fully understand the instructions before clicking the arrow to go on.  You **will not** be able to go back once you start.

-------

*[FIQ ITEM 1]*

What are all the different things this object could be?

**3. Convergent Creativity Task Instructions**

**Welcome to the Making Associations Task!**

You will be shown sets of three words at a time. For each set of three words, you will be asked to generate a fourth word that connects or fits with all three words. Specifically, the word that you generate, when combined with each of the three stimulus words, should create three word-pairs, each of which makes a common compound word or phrase. Please try to be **creative** and **appropriate** in your responses.

For example, one set of words that you might be shown is:

1. shelf

2. log

3. worm

A word that can be combined with each of these stimulus words is "book":

1. book shelf

2. log book

3. book worm

Another set of words that you might be shown is:

1. cream

2. skate

3. pick

A word that can be combined with each of these words to create a common word or phrase is "ice":

1. ice cream

2. ice skate

3. ice pick

Please provide your answers as quickly as you can. If you cannot think of a response for a particular set, please move onto the next set. Write down only the fourth word (i.e., "book" and "ice") as your answers. You will have 4 minutes for this task. After 4 minutes, the next page will automatically appear. Please make sure that you fully understand the instruction before clicking Next. You will not be able to return to this page.

**Complete the Analogy!**

**Instructions**

In this task you will be presented with word pairs.  Each word pair can form a coherent analogy, but part of the analogy is missing and is shown as a blank.  Your task is to provide the missing word. 

Please read the first word pair for each item, then provide a word in the blank space for the second word pair of the item that creates a coherent analogy.  An analogy is a logical connection or comparison between two things that might otherwise appear unrelated.  Please read the following examples of analogies as well as their subsequent answers and explanations.

***Example:***
**bear:cave :: bird:____**

This set of pairs would be connected analogically by answering with words such as “nest” or “tree” since this is a comparison between the homes of two different animals.  A bear lives in a cave and a bird lives in a nest.  Therefore, a bear is to a cave as a bird is to a nest, creating a coherent analogy.

Here is another example of an analogy:

**storm:hurricane :: relative:uncle**

This is a valid analogy because a hurricane is a type or kind of storm, and an uncle is a type of relative.

Please provide analogical answers to the following items.  Do not spend more than a few moments on each item, and avoid overthinking your response.  You will have 4 minutes for this task. After 4 minutes, the next page will automatically appear. Please try to provide a guess if you are struggling to find an answer.

**4. Supplemental Results: Correlations of Trait-Based Measures of Curiosity and Openness-to-Experience to Creative Ideation**

Table S1 presents the correlations between the three key trait-based measures of curiosity – the Curiosity subscale from Woo et al.'s [25] Openness to Experience questionnaire, and the Joyous Exploration and Deprivation Sensitivity subscales from the Five-Dimensional Curiosity Scale-Revised [47] – with our creative ideation measures.

**Table S1 Correlation of trait-based self-reported curiosity measures with creative ideation measures.**

| **Creative Ideation Measure** | **Correlation with**  **Curiosity subscale of**  **Woo Openness** | **Correlation with**  **Joyous Exploration**  **subscale** | **Correlation with**  **Deprivation Sensitivity subscale** |
| --- | --- | --- | --- |
| **Divergent Thinking Tasks** |  |  |  |
| AUT Fluency | *r* = .16, *p* = .031*  [.02, .30] | *r* = .08, *p* = .30  [-.07, .22] | *r* = .12, *p* = .13  [-.03, .26] |
| AUT Flexibility | *r* = .12, *p* = .12  [-.03, .26] | *r* = .08, *p* = .30  [-.07, .22] | *r* = .14, *p* = .08^  [-.01, .28] |
| AUT Originality | *r* = .22, *p* = .003**  [.08, .36] | *r* = .17, *p* = .023*  [.02, .31] | *r* = .15, *p* = .045*  [.003, .29] |
| FIQ Fluency | *r* = .06, *p* = .42  [-.09, .21] | *r* = .03, *p* = .70  [-.11, .18] | *r* = -.02, *p* = .84  [-.16, .13] |
| FIQ Flexibility | *r* = .05, *p* = .51  [-.10, .20] | *r* = .02, *p* = .82  [-.13, .16] | *r* = -.05, *p* = .55  [-.19, .10] |
| FIQ Originality | *r* = .11, *p* = .16  [-.04, .25] | *r* = .08, *p* = .28  [-.07, .23] | *r* = -.02, *p* = .78  [-.17, .13] |
| **Convergent Thinking Tasks** |  |  |  |
| RAT Proportion Correct | *r* = .02, *p* = .75  [-.12, .17] | *r* = .09, *p* = .24  [-.06, .23] | *r* = -.01, *p* = .95  [-.15, .14] |
| Analogy Completion  Proportion Correct | *r* = .15, *p* = .049*  [.001, .29] | *r* = .18, *p* = .018*  [03., .32] | *r* = .04, *p* = .58  [-.11, .19] |

*Note: *** p < .001, ** p < .01, * p < .05, ^^^ p < .10.* All *p*-values are two-tailed. AUT = Alternative Uses Task;

FIQ = Figural Interpretation Quest; RAT = Remote Associates Task; Analogy Completion is for the semantically distant analogies. Correlations are reported for comprehensiveness and to allow comparisons with previous studies examining self-reported curiosity with creative ideation.

From Table S1, it can be seen that both the curiosity subscale of the Woo openness to experience measure and the Joyous Exploration subscale modestly positively correlated with particularly the originality of participant's responses on the AUT, and also with the proportion of correct answers they generated on the Analogy Completion task. The Deprivation Sensitivity subscale correlated with the originality of participant's responses on the AUT, but with no other measures.

Given the well-established association between Openness to Experience and Creative Ideation [23, 24], to allow examination and comparisons of specifically the contribution of the curiosity component of Openness to creative ideation, Supplementary Results Table S2 gives the correlations between the Global measure of Openness (that does not separately consider curiosity but simply incorporates it together with all of the other facets into the overall score), and also the intermediate aspects of Intellect (which includes Efficiency, Ingenuity, and Curiosity) and Culture (which includes Aesthetics, Tolerance, and Depth) with each of the Creative Ideation Measures.

From Table S2 it can be seen that both AUT Originality and FIQ Originality significantly positively correlate with Global Openness and also with the Intellect aspect of this broad personality trait; additionally, AUT Originality also correlates with the Culture aspect. The predominantly convergent Analogy Completion task likewise significantly positively correlates with Global Openness and with the Intellect aspect. Although these positive associations are also observed when confining consideration to the Woo Curiosity scale (compare with Table S1), the associations appear to emerge more strongly when considering the Global Openness and Intellect aspects, with this stronger pattern observed for all three measures: AUT Originality, FIQ Originality (which was not significant for the Woo Curiosity scale on its own) and Analogy Completion. Tentatively (and while recognizing that there are differences in the number of items contributing to these comparisons) this suggests that, although curiosity on its own is an important contributor to the often-observed link between openness to experience and creative ideation, other cognitive-motivational characteristics that are associated with the complex trait of openness to experience, such as Ingenuity and Efficiency, may also contribute to that linkage.

**Table S2 Correlation of trait-based openness to experience measures with creative ideation measures.**

| **Creative Ideation Measure** | **Correlation with**  **Woo Global Openness** | **Correlation with**  **Woo Intellect aspect** | **Correlation with**  **Woo Culture aspect** |
| --- | --- | --- | --- |
| **Divergent Thinking Tasks** |  |  |  |
| AUT Fluency | *r* = .21, *p* = .005**  [.06, .35] | *r* = .13, *p* = .079^  [-.02, .27] | *r* = .22, *p* = .003**  [.08, .36] |
| AUT Flexibility | *r* = .11, *p* = .13  [-.03, .26] | *r* = .07, *p* = .36  [-.08, .22] | *r* = .13, *p* = .097^  [-.02, .27] |
| AUT Originality | *r* = .22, *p* = .003**  [.08, .36] | *r* = .19, *p* = .01*  [.05, .33] | *r* = .19, *p* = .012*  [.04, .33] |
| FIQ Fluency | *r* = .11, *p* = .14  [-.04, .25] | *r* = .12, *p* = .12  [-.03, .26] | *r* = .08, *p* = .32  [-.07, .22] |
| FIQ Flexibility | *r* = .13, *p* = .08^  [-.02, .27] | *r* = .12, *p* = .11  [-.03, .26] | *r* = .11, *p* = .16  [-.04, .25] |
| FIQ Originality | *r* = .16, *p* = .038*  [.01, .30] | *r* = .16, *p* = .03*  [.02, .30] | *r* = .11, *p* = .16  [-.04, .25] |
| **Convergent Thinking Tasks** |  |  |  |
| RAT Proportion Correct | *r* = .10, *p* = .17  [-.04, .25] | *r* = .12, *p* = .11  [-.03, .26] | *r* = .14, *p* = .061^  [-.01, .28] |
| Analogy Completion  Proportion Correct | *r* = .23, *p* = .002**  [.08, .36] | *r* = .26, *p* < .001***  [.11, .39] | *r* = .14, *p* = .068^  [-.01, .28] |

*Note: *** p < .001, ** p < .01, * p < .05, ^ p < .10.* All *p*-values are two-tailed. AUT = Alternative Uses Task;

FIQ = Figural Interpretation Quest; RAT = Remote Associates Task; Analogy Completion is for the semantically distant analogies.

**5. Supplemental Results: Effects of Task Order on the Curiosity Q&A Task**

The study was completed entirely online (via Qualtrics). It employed a within-subjects experimental design, with all tasks and measures administered to all participants. There was one between-subjects factor of task order, which manipulated the order of the three main behavioral measures, including the Curiosity Q&A task, the Divergent thinking tasks, and the Convergent thinking tasks. There was an approximately equal number of participants in each of the three task orders of administration: Order A (Informed Consent, Q&A, Divergent, Convergent, Questionnaires, *n* = 61); Order B (Informed Consent, Divergent, Q&A, Convergent, Questionnaires, *n* = 56); Order C (Informed Consent, Convergent, Q&A, Divergent, Questionnaires, *n* = 62).

**Effects of Task Order on Question-Asking**

Table S3 presents the number of Novel Questions asked by participants in each of the three task orders.

**Table S3.**

Average Novel Qs SD

Q&A was the first task (Order A) .39 .62

Q&A was after divergent tasks (Order B) 1.14 1.75

Q&A was after convergent tasks (Order C) .56 .72

Participants asked significantly more Novel Qs if they completed the Q&A Task after the Divergent Thinking Tasks, *F*(2, 176) = 6.95, *p* = .001, with post-hoc tests (not assuming equal variance) showing Order B significantly higher than Order A. This pattern was also observed when using a nonparametric test comparing the medians of the three orders: Order A vs. Order B, *p* < .001.


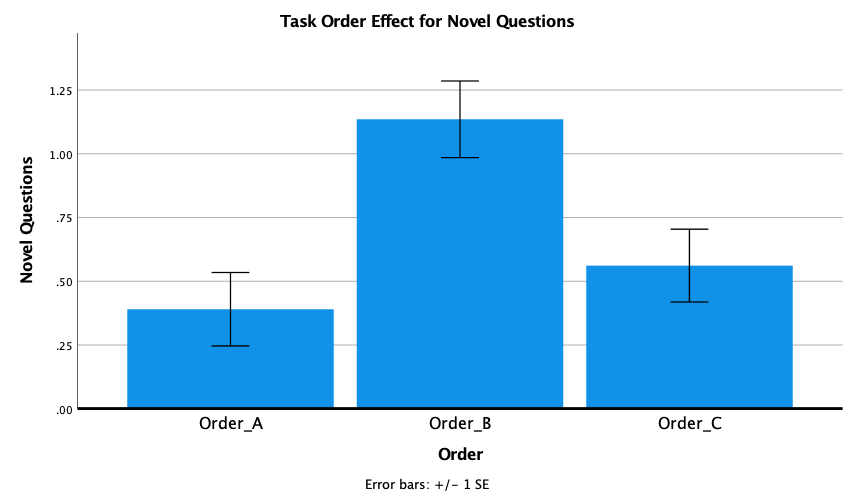


Table S4 presents the number of Topic-Related Questions asked by participants in each of the three task orders.

**Table S4.**

Average Topic-Related Qs SD

Q&A was the first task (Order A) 1.07 1.24

Q&A was after divergent tasks (Order B) 2.47 2.37

Q&A was after convergent tasks (Order C) 1.45 1.37

A similar pattern was observed for Topic-Related Questions: Participants asked significantly more Topic-Related Qs if they completed the Q&A Task after the Divergent Thinking Tasks, *F*(2, 176) = 10.38, *p* < .001, with post-hoc tests (not assuming equal variance) showing Order B significantly higher than Order A. This pattern was also observed when using a nonparametric test comparing the medians of the three orders: Order A vs. Order B, *p* < .001, and also Order B vs. Order C, *p* = .003.


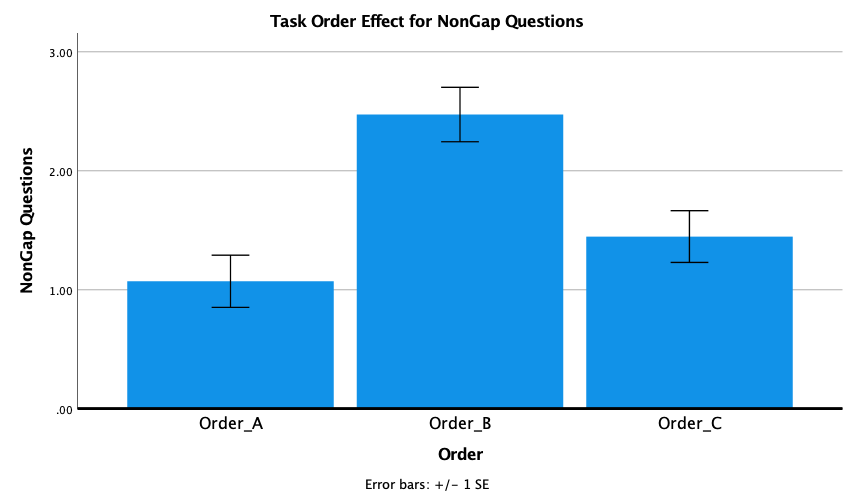

Supplement: Supplementary file 1 — Supplementary Information. [file 41598_2022_19694_MOESM1_ESM.docx]
